# Clinical Characterisation and Management of the Main Treatment-Induced Toxicities in Patients with Hepatocellular Carcinoma and Cirrhosis

**DOI:** 10.3390/cancers13030584

**Published:** 2021-02-02

**Authors:** Fausto Meriggi, Massimo Graffeo

**Affiliations:** 1 Oncology Department, Istituto Ospedaliero Fondazione Poliambulanza, Via Leonida Bissolati 57, 25124 Brescia, Italy; 2 Hepatology Unit, Istituto Ospedaliero Fondazione Poliambulanza, Via Leonida Bissolati 57, 25124 Brescia, Italy; massimo.graffeo@poliambulanza.it

**Keywords:** hepatocellular carcinoma, locoregional treatments, systemic treatments, toxicities

## Abstract

**Simple Summary:**

The incidence of hepatocellular carcinoma continues to increase worldwide. In almost all cases, hepatocellular carcinoma develops in subjects with hepatic cirrhosis and patients can therefore present symptoms that are attributable to both conditions. There are several ablation techniques currently available for the treatment of unresectable HCC associated with early-stage cirrhosis. Moreover, novel therapies with biological agents and immunotherapy have come to be standard options in the approach to systemic treatment of hepatocellular carcinoma. However, in addition to being costly, these drugs are not devoid of adverse effects and their management cannot forgo the consideration of the underlying hepatic impairment. Therefore, these patients require a mandatory multidisciplinary management.

**Abstract:**

The incidence of hepatocellular carcinoma (HCC) continues to increase worldwide, particularly in Western countries. In almost all cases, HCC develops in subjects with hepatic cirrhosis, often as the result of hepatitis B or C virus infection, alcohol abuse or metabolic forms secondary to non-alcoholic steatohepatitis. Patients with HCC and hepatic symptoms can therefore present symptoms that are attributable to both conditions. These patients require multidisciplinary management, calling for close interaction between the hepatologist and the oncologist. Indeed, the treatment of HCC requires, depending on the disease stage and the degree of hepatic impairment, locoregional therapies that can in turn be broken down into surgical and nonsurgical treatments and systemic treatments used in the event of progression after the administration of locoregional treatments. The past decade has seen the publication of countless papers of great interest that have radically changed the scenario of treatment for HCC. Novel therapies with biological agents and immunotherapy have come to be standard options in the approach to treatment of this cancer, obtaining very promising results where in the past chemotherapy was almost never able to have an impact on the course of the disease. However, in addition to being costly, these drugs are not devoid of adverse effects and their management cannot forgo the consideration of the underlying hepatic impairment. Patients with HCC and cirrhosis therefore require special attention, starting from the initial characterisation needed for an appropriate selection of those to be referred for treatment, as these patients are almost never fit. In this chapter, we will attempt to investigate and clarify the key points of the management of the main toxicities induced by locoregional and systemic treatments for HCC secondary to cirrhosis.

## 1. Introduction

The incidence of HCC continues to increase worldwide, especially in Western countries, where its presence is closely related to that of hepatic cirrhosis [1]. In almost 80% of cases, the tumour develops in a liver that has undergone the evolutionary effects of anatomic remodelling and a transformation that have led to the development of cirrhosis. In addition, the aetiology that caused and influenced this evolution is very often associated with underlying hepatitis B or C infection, as well as alcohol abuse and metabolic conditions secondary to non-alcoholic steatohepatitis (NASH) [2]. This creates a diverse stratification not only of the aetiological factors underlying the transformation process and their consequent management, but also of the treatment of the tumour and the factors influencing prognosis. The prognosis for this kind of tumour has changed greatly over the last two decades with the advent of novel treatment options associated with the screening programmes provided to patients with cirrhosis that make it possible to identify HCC at an early stage, and therefore to implement potentially increasingly curative treatments. The treatment options for HCC can be broken down into surgical therapies (i.e., resection, cryoablation and liver transplantation) and nonsurgical therapies that can target the liver and are therefore termed “locoregional” (such as, for example, percutaneous ethanol injection, radiofrequency/microwave thermal ablation, transarterial chemoembolisation, external beam radiotherapy) or systemic treatments (chemotherapy, targeted therapy, immunotherapy with checkpoint inhibitors). In some cases, it is somewhat difficult to assess the benefits in terms of survival of a systemic therapy regimen in patients with advanced HCC, as survival is often determined not so much by the aggressiveness of the tumour or by the impact of a systemic treatment, but primarily by the degree of hepatic dysfunction generated by the liver disease. Systemic chemotherapy is not usually well tolerated by these patients with advanced HCC and its use has a limited scope, also in view of the rather disappointing results. Moreover, for patients with Child-Pugh class C cirrhosis and those with an impaired performance status (PS) or severe comorbidities, supportive care alone still appears to be the most indicated option. Prior to 2008, there was no truly efficacious systemic therapy for patients with advanced HCC or those who were refractory to locoregional therapies. There has been a consequent renewed interest in and enthusiasm for systemic therapy for HCC with the publication of data showing that the targeted agents sorafenib [3,4] and regorafenib [5] were able to improve survival compared to the best supportive therapy. Subsequently, the benefit in terms of survival was demonstrated also for second-line therapy with nivolumab [6,7,8], an immune checkpoint inhibitor, and lenvatinib [9] which was seen to be non-inferior to sorafenib as first-line therapy. Systemic therapy is now considered to be appropriate for patients with unresectable advanced HCC who are not eligible for or who have progression following locoregional treatment and whose liver function is likely to be able to tolerate the therapy (e.g., Child-Pugh class A or B7 cirrhosis). According to the most recent National Comprehensive Cancer Network (NCCN) guidelines [10], there are currently three systemic therapy options that can be used for first-line treatment (in chronological order of approval): sorafenib [3,4], lenvatinib [9,11] and the more recent atezolizumab plus bevacizumab combination [12]. In those patients who are not eligible for treatment with a tyrosine kinase inhibitor (TKI), immunotherapy with nivolumab can be considered [13]. At progression, in those patients who maintain Child-Pugh class A or, for certain agents, also class B7 liver function, and depending on the administered first-line treatment, there are a number of systemic therapy options: regorafenib (5), cabozantinib [14], ramucirumab [15], lenvatinib or immunotherapy with nivolumab [6,7,8], nivolumab plus ipilimumab [16], pembrolizumab [17], or sorafenib.

## 2. Staging

Given the close relationship between HCC and cirrhosis, the classic staging systems commonly used in oncology are inadequate for obtaining correct prognostic stratification and an appropriate consequent allocation of therapy, given the co-existence of two potentially fatal conditions. For this reason, in the late 1990s, a new staging system was devised using evidence-based medicine (EBM) data, the BCLC (Barcelona Clinical Liver Cancer staging system) [18]. This system, which is now applied in most of the world, takes into consideration the stage of the tumour (dimensions, vascular invasion, distant metastases), but also liver function according to the classic stratification using the patient’s Child-Pugh and PS scores. The indisputable advantage of this classification system is that it provides, for each given disease stage, a well-defined treatment option depending on the patient’s life expectancy. Although all of these systems have limits, the information they provide for distinguishing patients with maintained liver function from those with advanced liver disease is crucial in terms of prognosis and treatment decision-making. As an alternative to the Child-Pugh classification system, the Model for End-stage Liver Disease (MELD) or the Albumin-Bilirubin (ALBI) score can be used [19,20]. This has made it possible, for example, to restrict the resection criteria for HCC, which were once associated with high mortality due to decompensation, for patients with small tumours, low bilirubin levels and mild or no portal hypertension (PH). By maintaining these criteria, long-term survival is approximately 70% at 5 years. The criteria for transplantation have also been narrowed to patients with <3 nodules and diameter <3 cm, making it possible to reduce the risk of post-OLT recurrence to less than 15%. The best results in terms of survival (75–80% at 5 years) after liver transplantation for HCC secondary to cirrhosis are obtained, in fact, in patients who meet the “Milan criteria” (i.e., a single nodule ≤5 cm or no more than 3 nodules with a diameter ≤3 cm) [21,22,23]. The prognosis of patients with HCC secondary to cirrhosis remains good with liver transplantation, as it simultaneously provides treatment for the disease and the tumour. However, this treatment option is restricted by the limited number of donors and by specific contraindications (old age and comorbidities). All this information has improved the selection of elegible patients for percutaneous therapies and their risk stratification by identifying the ideal candidates, consequently improving their survival, for which the median values are very similar to those obtained with resection [21]. The intermediate stage refers to those patients who will undergo chemoembolisation or radioembolisation, whereas in the advanced stage, thanks to the ever-greater knowledge regarding the molecular processes involved in the pathogenesis of liver tumours, proliferation and angiogenesis, we are witnessing a continuous development of antitumour molecules against new, more specific biological targets, which have revolutionised the approach to patients with HCC. Another recent acquisition was the concept of “the stage migration strategy”, an approach by which a recommended treatment option for a different disease stage is offered to patients whose condition and tumour site make them ineligible for the gold standard first-line treatment on the basis of the BCLC staging system. All the strategies available require a multidisciplinary management, involving hepatologists, oncologists, radiologists, interventional radiologists, pathologists and surgeons, as well as general practitioners and psychologists [24,25].

## 3. Functional Assessment and Risk Stratification

**Figure 1:**
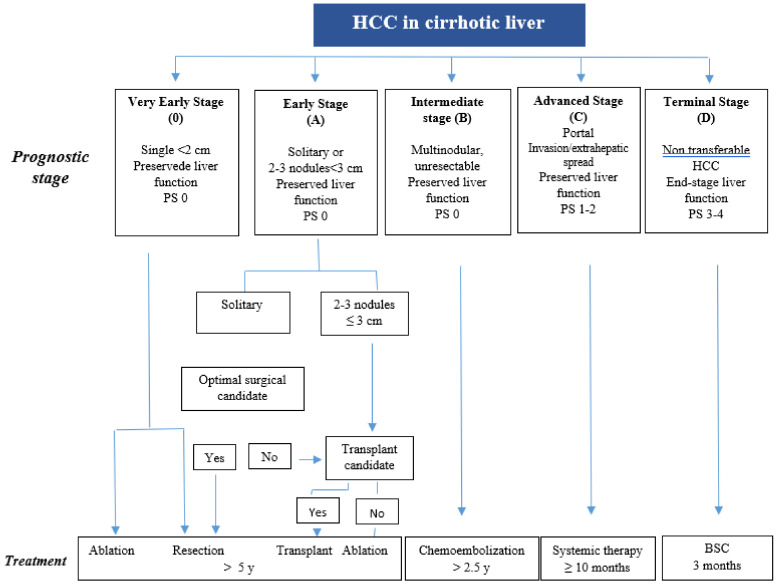
EASL Clinical Practice Guidelines: Management of hepatocellular carcinoma (J Hepatology 2018;69:182–236).

HCC, in its very early stage, is heterogeneous both in terms of liver function (i.e., presence or absence of PH, Model for End stage Liver Disease score, Child Pugh score 5 or 6, bilirubin level) and tumour characteristics (i.e., location, alpha-fetoprotein values, pathological features such as micro-vascular invasion, tumour grade and satellitosis). In the case of liver disease, especially advanced disease with HCC, the selection of the patients to be treated is a crucial part of the therapy itself and, drawing on the cancer staging experience acquired for other types of cancer, it must provide simple, reproducible and efficacious information in order to identify the prognosis, possible natural history and most effective treatment [21] (Figure 1). In Italy, the majority of cases of HCC occurs in patients with hepatic cirrhosis, with varying degrees of liver failure. Consequently, the prognosis of patients with HCC is influenced not only by the extent of the tumour, but also by the residual hepatic function. In patients with HCC associated with cirrhosis, the main problem is therefore that it is necessary to simultaneously consider two potentially fatal conditions. In the most commonly used staging system, prognosis is related to certain coexisting factors that can be evaluated for appropriate treatment decision-making and represents the first step in appropriate patient selection. The three factors in question are: ECOG PS [26,27]; dimensional and numerical tumour staging, which is similar to the TNM system usually used on oncology and obtained using imaging techniques, but that does not take into account the residual liver function; and the degree of cirrhotic compensation using the Child-Pugh score [28] (Table 1). This system has been used a great deal over the past 30 years and is based on both biological parameters (bilirubin, albumin, INR) and clinical ones (encephalopathy and ascites). Despite the limit represented by the subjectivity of the last two parameters, this score provides a reliable estimate of the survival of patients with cirrhosis alone. Although over time various staging systems (Okuda, Clip score, Gretch, Cupi) have attempted to formulate scores taking into account the three fundamental parameters (tumour staging, patient PS and residual liver function), the system that is most widely recognised in literature and in study protocols for new treatments is still the BCLC staging system. Some of the critical points that have nevertheless emerged regarding this staging system over time are: the difficulty in allocating optimum treatment to Child-Pugh class B patients, excessively vague intermediate grade, and the indication for systemic treatments only for advanced-stage patients. The recently devised ITA.LIC.A system validated on populations of different ethnic origins (Italian and Taiwanese) and with different tumour aetiologies has shown the greatest prognostic accuracy amongst the most commonly used systems [1,29,30].

## 4. Implications of Cirrhosis That Influence the Choice of Treatment

The points raised above show that the natural history of HCC and cirrhosis are closely related and inevitably intertwined. This makes it a difficult and complex task to clearly define not only the prevalence of the symptoms and complications experienced by patients, but also their exact origin, namely whether they can be attributed to the natural evolution of the cirrhosis or the HCC.

As selective as it may be, any surgical, percutaneous or locoregional treatment involves a certain degree of loss of functional hepatic tissue and, whereas in Child-Pugh class A patients it may be negligible and without serious sequelae, it can progress to more or less severe transient hepatic decompensation in Child-Pugh class B patients. In the worst functional class, Child-Pugh class C, in which failure is already present, all treatments are contraindicated with the exception of transplantation, provided the patient is eligible.

Consequently, the correct treatment of HCC necessarily requires a knowledge of the most common complications of cirrhosis and their respective treatment.

Presence or absence of PH, which is also able to cause a systemic circulation disorder that usually only becomes clinically overt during the ascitic decompensation phase. In this scenario, the most clinically relevant segment is the kidneys, where arterial vasoconstriction occurs with consequent sodium and water retention of varying degrees. The MELD or ALBI scores can be used as an alternative to the Child-Pugh score [20,31]. As the oncological treatment for HCC can cause albeit temporary changes in portal pressure, all patients with HCC secondary to cirrhosis should have an oesophageogastroduodenoscopy (EGDS) before starting therapy in order to identify any signs of portal hypertension and in order to rate the risk of variceal bleeds. The finding of oesophageal varices and/or splenomegaly with a platelet count <100 × 10^9^/L indicate the presence of “clinically-significant” PH (CSPH) and is useful in terms of prognosis and management. The presence of CSPH can be ruled out in a non-invasive manner using the Baveno VI criteria (platelet count >150 × 10^9^/L or liver stiffness of <20 kPa76 on the elastography). In the case of oesophageal varices with a high risk of rupture, bleeding prevention therapy must be initiated (with betablockers or elastic band ligation through to eradication) [32,33].

Clinically relevant PH and high bilirubin levels are associated with high mortality and morbidity following local resection (LR) (EASL). However, as reported by the ITA.LI.CA Group, LR may be extended to patients with either clinically significant PHT or slight hyperbilirubinemia (<2.0 mg/dL) without compromising outcomes [34].

Tumour location, need for extensive or complex liver resection, local donor resources and waiting list pressure are some examples of the important variables that need to be considered among patients with HCC [35] for example nodules with a deep/central location in the liver usually require a technically complex LR, while surface nodules can be easily resected using a minor LR particularly when located in the anterior segments of the liver [36].

Another critical point is that more and more often the patients in real live are older than in the past and the need of the knowledge of all the comorbidity could change the risk stratification and the priority of treatment. Even if many different comorbidity scores are still existing, their applicability in the field of HCC are lacking up to now.

A study from Italy performed on 919 HCC-on-cirrhosis consecutive patients undergoing LR showed that postoperative mortality and 3-year survival rates were similar among age quartiles (≤60, 60–66, 67–70 and >70 years) [37].

## 5. Locoregional Therapies

The ablation techniques currently available for the treatment of unresectable HCC associated with early-stage cirrhosis (BCLB-0) are (Table 2). In general, patients with very early HCC who are treated with any of these strategies can have excellent recurrence free and overall survival outcomes compared with patients who have more advanced tumours.

### 5.1. Radiofrequency Thermal Ablation (RFA)

The standard of care for the treatment of early forms, including as a first-line option if the tumour is conveniently located even in patients who are eligible for resection. The major complications in cirrhotic patients are thermal damage to the adjacent tissues, the heat dissipation effect close to large blood vessels and the risk of perforation close to hollow organs or in the presence of adhesion syndrome for superficial lesions. Locoregional RFA is indicated depending on the BCLC stage in a position downstream of the resection; however, in the case of multidisciplinary decision-making, it can be considered as first-line therapy for single nodules with a diameter of up to 2 cm, as, compared to surgical resection, it is associated with lower morbidity and it involves shorter hospitalisation times and lower costs with similar survival. For nodules measuring between 2 cm and 3 cm, the choice between resection and RFA must always be made by a multidisciplinary team on the basis of the patient’s characteristics and the lesion site. For nodules >3 cm, resection should be chosen whenever possible. The choice of treatment for small single nodules (<3 cm) is based on randomised studies with suboptimum sample sizes and study design. At the current time, the choice between surgery and RFA in these patients is based on the existence of comorbidities, the visibility of the nodule on the ultrasound scan and technical considerations (location of the lesion and its vicinity to structures potentially at risk of RFA-induced damage (e.g., stomach, hepatic flexure, gallbladder, bile ducts) [38,40]. In the case of a single nodule with a diameter of up to 2 cm, RFA can be the most cost-effective option, as it is able to achieve complete necrosis of the lesion in 98% of cases with lower direct costs, shorter hospitalisation times and lower morbidity rates than resection, and has a negligible mortality risk [41], (ruling out nodules that are superficial and situated close to large blood vessels or the gallbladder).

### 5.2. Microwave Ablation (MWA)

Has shown promising results. Despite being more rapid and the higher local temperature level, there is no reliable indication as to how much energy should be applied and which local inconveniences can be expected. MWA has been part of routine clinical practice for some years now and the available data seem to suggest that for the treatment of nodules >3 cm it is superior to RFA in obtaining complete necrosis and has a longer progression-free survival interval with a similar safety profile [42]. MWA was seen to have a significantly better local recurrence rate (OR 2.21, 95% CI: 1.19–4.07, *p* < 0.01) in a recent meta-analysis of comparative studies (4 RCTs and 10 observational studies) [43]. One RCT comparing RFA with MWA has been published and it suggests that the two techniques are unable to offer a true advantage in terms of overall survival in cirrhotic patients with early-stage HCC, although it does suggest that better local control is achieved with MWA [44]. The choice between the two methods would therefore appear to be dictated by logistical rather than clinical considerations, as the costs are very similar. A non-negligible number of nodules are not visible on the ultrasound or they are situated in risk positions (visceral organs, gallbladder). In these situations, video laparoscopy is a valid alternative.

### 5.3. Ethanol Injection (Alcoholisation)

Still represents a feasible option in those cases in which, due to its location, the tumour cannot technically be treated using RFA, especially for tumours <2 cm. (evidence quality: high; recommendation: strong). Although percutaneous ethanol injection (PEI) produces poorer results than RFA, it can be used in 10–15% of patients with HCC ≤2–3 cm in sites that are considered to be at risk with RFA treatment [45,46].

### 5.4. Cryotherapy

Is an option that is currently being validated, as is irreversible *electroporation,* and little data and few clinical studies are available for these techniques.

Combination treatments. When the diameter of the nodule exceeds 3.5 cm, in patients who are not eligible for resection and who have a good hepatic functional reserve, it is reasonable to consider using combined or sequential treatments (chemoembolisation + RFA or PEI) as an alternative to MWA or RFA with multiple insertions. The most common combination treatment and that for which the most literature data is available is TACE + RFA. The data of a meta-analysis performed on 8 RCTs showed the superiority of combined therapy vs. RFA alone in terms of both OS and RFS (recurrence-free survival), especially in the case of larger tumours (>3 cm) [46].

### 5.5. Stereotactic Body Radiotherapy (SBRT)

Radiation therapy is considered for patients with liver function too poor for liver directed interventional radiology procedures, for patients who have failed such therapies, or for those with medical comorbidities that preclude them from undergoing such procedures. SBRT is indicated especially in cases in which RFA is insufficient for obtaining local disease control due to the site of the nodule to be treated [47]. The eligibility criteria for SBRT treatment usually include tumour size ≤6 cm and a number of lesions ≤3.

To prevent hepatic decompensation, the residual hepatic function would be Child-Pugh class A or ≤B7, absence of ascites, tumour-free liver volume >700/1000 cc. All treatment decision-making should be validated by a multidisciplinary team. Compared to conventional radiotherapy, SBRT makes it possible to concentrate in the target lesion higher fractionated doses of radiation whilst preserving the adjacent liver tissue from exposure with an undisputable advantage in terms of safety. In the liver cancer field, increasing attention is currently being dedicated to the use of radiotherapy in combination with TACE in those cases in which TACE alone is ineffective [48].

### 5.6. Superselective Chemoembolisation (TACE)

In selected patients has been seen to be particularly efficacious in BCLC-B patients. The use of DEB (drug-eluting bead)-TACE achieves similar results to conventional TACE, but may have fewer side effects. TACE should not be used in patients with hepatic decompensation (>Child-Pugh class B7), advanced liver and kidney dysfunction, macroscopic vascular invasion and extrahepatic disease. The common rationale of TAE and TACE lies in causing primarily ischaemic damage to the tumour cells by means of the superselective occlusion of the arterial vessels afferent to the tumour. Furthermore, in TACE (cTACE or Deb-TACE) a chemotherapy agent is added. To date no prospective study has demonstrated the superiority of conventional TACE over embolisation alone, embolisation with drug-eluting beads or radioembolisation. It is worth mentioning a randomised study by K.T Brown with particularly well-designed technical aspects in which 101 patients with HCC were randomised to receive embolisation with microspheres loaded with 150 mg of Doxorubicin (LC) vs. unloaded microspheres (Bead Block). The comparison between the two study arms did not show any significant difference in terms of either RECIST radiological response or in terms of survival (OS), 19.6 versus 20.8 months (HR 1.11; 95% CI: 0.71–1.76; *p* = 0.64). Other randomised studies comparing conventional TACE and DEB-TACE did not show the latter to be superior in terms of either antitumour activity or survival [49,50].

The performance of TACE can be extended to Child-Pugh class B patients with a score of 7 in the absence of ascites. TACE can be repeated at regular intervals (usually every 2 months, until complete response and in any case no more than 3 times on the same nodules) or “on demand”, according to the response to the previous treatment. This latter approach would appear to be associated with fewer complications. In the case of two-lobe disease and when it is not possible to perform superselective treatment on the various lesions, the treatment of each lobe should be performed in sequential sessions at least 1 month apart, provided the patient’s clinical condition does not deteriorate. TACE must be discontinued in the case of complete response, no response (progression and stability) by the target lesions after 2–3 procedures and, of course, if serious adverse events occur. As TACE can result in tumour necrosis that is not necessarily associated with a reduction in the size of the HCC, when assessing the response to TACE it is advisable to use criteria that take into account the necrosis induced by the treatment and not simply the variations in size, such as the modified RECIST criteria or the EASL criteria that evaluate the portions of the tumour that remain perfused [51] as indicated in the structured report template proposed by the SIRM.

TACE can cause end-stage liver disease in approximately 2% of cases, even when patients are well selected. Current treatment-related death is estimated to be less than 1% in patients with HCC [52]. The most common adverse events were related to the postembolization syndrome, and included liver enzyme abnormalities (18.1%), fever (17.2%), abdominal pain (11.0%), vomiting (6.0%), and nausea (1.7%). A short course of steroids was shown to reduce the incidence of postembolization syndrome in some reports [53].

Following TACE, signs of acute liver injury are commonly seen. Miksad et al. assessed the liver function deterioration in a retrospective, observational study, 30–90 days after a single TACE, in real-world practice. A significant proportion of HCC patients had deterioration of liver function (23% to 30%) [54]. The careful selection of patients for TACE is therefore crucial because liver dysfunction may preclude other systemic therapy options.

### 5.7. Radioembolisation (TARE) with Yttrium 90

In the more advanced liver surgery Centres, TARE is increasing frequently used to obtain “downstaging” and “downsizing” in patients who were initially not eligible for resection or transplantation according to the Milan criteria.

TARE/SIRT with Yttrium-90 were studied in BCLC-A patients as bridging therapy to transplantation, BCLC-B compared with TACE and BCLC-C compared with Sorafenib. The data show a good safety profile and good local control, but no improvement in terms of survival compared to sorafenib alone in BCLC-B and C patients.

A number of retrospective studies have reported the safety and efficacy of TARE in patients with HCC, with or without neoplastic thrombotic invasion of the portal circulation. A recent study reported a considerable difference in median OS with TARE compared to sorafenib (26.2 vs. 8.7 months, *p* = 0.054) in patients with HCC and portal thrombosis [55]. Treatment with TARE is also associated with better overall survival (HR 0.40 [0.19–0.82]; *p* = 0.013). Furthermore, grade 3–4 adverse events were more common with Sorafenib than in the TARE arm (44.6 vs. 17.6%) [55]. TARE can represent a valid treatment option for patients with recurrence after surgery, thermal ablation treatments or intravascular procedures [56], although in the two randomised multicentre phase 3 studies conducted on two different populations in France and Asia, respectively (SARAH and SIRveNIB), TARE did not show better survival than Sorafenib despite demonstrating radiological response of approximately 20% vs. 12% for Sorafenib.) [57,58]. In patients with intermediate HCC who are not eligible for locoregional treatment or those with advanced HCC due to portal vein thrombosis and without metastases in Child-Pugh class A, TARE can be considered as a first-intention treatment option especially for patients who are elderly or have comorbidities. The performance of TARE requires a high level of operator and centre specialisation and it cannot be administered in the presence of intrapulmonary shunting >20% or vascular abnormalities that may result in the irradiation of hollow organs (stomach and intestine) responsible for severe gastritis and ulcers [41,59].

Several studies suggest that combination treatments (TACE + RFA) increase tumour response to locoregional therapy by increasing the volume of tumour necrosis that can be obtained [60]. The most frequent side effects of TARE is the post-(radio)embolization syndrome with fatigue, nausea, vomiting, anorexia, fever and abdominal discomfort that may occur in up to 55% of patients and is self-limiting, lasting no longer than two weeks. An elevation of liver enzymes, alkaline phosphatase, alanine transferase and bilirubin are normal side effects of this treatment. The most common relevant complication of TARE is gastrointestinal (GI) ulceration and Proton pump inhibitors are useful to reduce the risk and represent the treatment of choice. Radiation induced liver disease, with comparison of jaundice, non-malignant ascites combined with an increase in alkaline phosphatase to at least twice the upper normal level within four months after treatment. It may occur in up to 20% of patients. To reduce the risk to this potential progressive dysfunctions is crucial the selection of patients by liver function and the administered radiation dose. The routine administration of ursodeoxycholic acid and low-dose steroids has been shown to significantly reduce the risk [61,62].

## 6. Main Adverse Events in Cirrhotic Patients

One of the main side effects to be considered in both treatment-naive patients and those being treated with antiangiogenetic agents and that can worsen the condition and management of the disease is diarrhoea. It is not an uncommon finding in patients with cirrhosis and HCC and is in part directly explained by the production by the tumour of secreted substances such as gastrin and vasoactive intestinal peptide. In addition, diarrhoea is a common adverse event of a number of therapies, including those used in both common oncological practice and HCC-specific practice (approximately 7% of all adverse drug reactions). It usually presents with a change in normal defaecation frequency and consistency whose detrimental effect perpetuates if the symptom becomes chronic. In the pivotal studies for Sorafenib, the first of the anti-angiogenetic agents to be included in the therapeutic formulary, the onset of diarrhoea ranged from 25.5% in the Asian-Pacific study to 39% in the SHARP study [3,4]. The correct characterisation of diarrhoea by means of common biochemical and culture tests and compliance with appropriate dietary advice avoiding restrictions and using medicinal products such as racecadotril, loperamide and octreotide for grade 1 and 2 toxicities usually reduces the risk of having to suspend the oncological treatment. Cancer-related fatigue would appear to be one of the most relevant symptoms in patients with cirrhosis; in many cases it exists even prior to therapy and tends to become worse over time, thereby impairing concentration and the quality and quantity of sleep. Frustration, irritability and sadness are exacerbated and there is an increase in the subjective feeling of physical tiredness that has a negative impact on the activities of daily living. The profound asthenia does not attenuate with rest and correlates with the disease and its treatment. It affects approximately 70–98% of patients with liver cancer and electrolyte imbalances, thyroid function, increased cytokine production, serotonin imbalances and vagal response activation all play a role in its aetiology. Elderly patients (>80 years) who are already debilitated before any treatment are those that are worst affected. In cirrhotic patients, fatigue is the first symptom to present in approximately 50% of cases. Treatment is multimodal and consists in causal therapy (for anaemia, fever, electrolyte imbalances, depression, etc.) and systemic therapies constituted by steroids, methylphenidate and modafinil. In cirrhotic patients, the scope for specific treatments is more limited and complicated due to the hepatotoxic effect of certain therapies and therefore patients must be motivated by focussing on the objectives of therapy by involving them in training activities without underestimating the mental and depressive aspects. Physical exercise and omega 3 dietary supplementation can make a valid contribution.

## 7. Exacerbation of HBV and HCV

There are currently a number of medicinal products available for the treatment of cirrhotic-stage HBV infection, most importantly those belonging to the nucleotide analogue category.

Patients with HBV replication induced cirrhosis (confirmed by the presence of viral DNA in the blood), complicated by HCC must be quickly treated with antiviral therapy for HBV with nucleoside/nucleotide analogues (Entecavir, Tenofovir disoproxil fumarate, Tenofovir alafenamide), especially when oncological treatments are to be administered. HBV virus can now be adequately controlled in terms of HBV DNA virology with the consolidated use of antivirals such as Tenofovir and Entecavir and exacerbations caused by the acquisition of resistance rarely occur during treatment [62].

However, in the case of HCV, even if severe exacerbation and/or decompensation of the chronic liver disease is currently uncommon, survival in hepatitis C virus (HCV) patients with cirrhosis and successfully treated HCC, is mainly influenced by early hepatic decompensation. In viraemic HCV patients with radiological complete response after treatment for HCC, therapy with direct-acting antivirals (DAAs) improves survival by reducing the risk of decompensation of the chronic liver disease [63]. Furthermore, where possible, eradication therapy is appropriate in cases of compensated cirrhosis without HCC through the use of these DAAs. In the case of HCC to be treated with locoregional therapy, treatment must be postponed once response to the surgical or locoregional treatment has been confirmed at least until after the first CT scan confirming that disease control has been achieved with the therapy. On the other hand, a number of meta-analyses have confirmed that, in viraemic patients, it is possible to obtain a benefit in terms of survival with antiviral therapy, due to its favourable effect on disease progression and non-HCC-related mortality. Furthermore, a recent multicentre study comparing an Italian cohort of 163 patients who were treated with DAAs after radiological complete response to the HCC treatment with 328 viraemic HCV patients also with HCC and complete response to the treatment, showed, for the first time an advantage in terms of survival in cases treated with DAAs vs. untreated patients (HR = 0.39; 95% CI: 0.17–0.91, *p* = 0.03). This advantage was attributed to the protective effect of DAAs on the progression of cirrhosis [64].

## 8. Systemic Treatments

In recent years, the results obtained with TKIs, antiangiogenetic agents and immunotherapy have radically changed the scenario for the treatment of advanced HCC and its prognosis, with positive repercussions on both PFS and overall survival. However, in addition to being very expensive and therefore posing sustainability problems, these agents are not devoid of side effects (Table 3). Consequently, there is probably still a role to be played by chemotherapy in certain selected patients who maintain adequate liver function and a reasonable residual life expectancy. One of the greatest difficulties encountered in the treatment of these patients is the clinical management of both the symptoms associated with the underlying chronic liver disease that almost always affects patients with HCC and the management of the side effects of the various locoregional and/or systemic treatments. More specifically, the management of adverse events is extremely complex because they are often the expression of a positive response to the on-going cancer therapy (especially in the case of targeted therapy) and therefore, insofar as is possible, they should not be considered grounds for an early discontinuation of the treatment. Among the adverse events (AEs) caused by TKIs, HFSR and diarrhoea are particularly detrimental for the patient’s quality of life (QoL). This calls for appropriate patient information and the prevention of the most common adverse events (Table 4). Many recommendations for managing HFSR are based on clinical experience rather than scientific evidence, however patients should use prophylactic emollients containing 10% urea and removed existing areas of hyperkeratosis before TKI treatment. For grade 2 or 3 HFSR, topic steroids are indicated. In the SHARP trial, the incidence of severe grade 3/4 diarrhoea was seen in 11% of enrolled patients in the sorafenib arm [3]. Those patients require supportive therapy and loperamide (Table 4). Hypertension is another common AE in TKI-treated patients. Usually, when blood pressure is ≥140/90, administration of an antihypertensive agent such as angiotensin-converting enzyme inhibitor (ACEi) or angiotensin II receptor blocker (ARB) is recommended. In this scenario, the multidisciplinary team therefore takes on a fundamental role, in particular the close partnership that should be developed between the oncologist and the hepatologist. Separate consideration should be dedicated to elderly patients with advanced HCC, because it requires special attention with regard to the administration of these therapies in order to slow the evolution of the liver tumour in patients with cirrhosis. These patients are often frail due to their comorbidities, unable to deal with any side effects and, therefore unable to receive any cancer treatment other than the best supportive care alone. A comprehensive assessment must be performed by analysing the functional reserve and life expectancy, using tools such as the Comprehensive Geriatric Assessment (CGA), which has proven efficacy in selecting those elderly patients who may obtain benefit from a given treatment. Nutritional status also represents an important aspect of the management of elderly subjects with HCC such as to prejudice the expected results of therapy and survival. One useful tool in such situations is the Mini Nutritional Assessment (MNA), which is able to identify those elderly subjects at risk of malnutrition. In a recent review, Arora et al. [65], observed that sorafenib in a population of elderly patients >70 years of age is poorly tolerated with a frequent need for dose reductions due to grade 3–4 toxicity or even a permanent discontinuation of the treatment [66,67,68,69,70,71]. The REFLECT study provided a retrospective investigation on the benefit of treatment with Lenvatinib, which was seen to be similar in the group of patients <65 years and in those >65 years with grade 3–4 toxicity and a lower treatment discontinuation rate than was recorded with Sorafenib [9]. HCC treatment with immunotherapy deserves a chapter of its own. The inhibition of the physiological immune checkpoints exerted by these agents can be associated with immune-related adverse events (irAEs) (Table 5 and Table 6) that can involve any organ and system, but especially the skin, intestine, thyroid, adrenal glands, lungs and liver [6]. The most common immune-mediated AEs are similar to those with other tumor types, but the rate of hepatitis may be slightly higher. Most relevant, toxicities should be recognized early on and addressed appropriately. For monotherapy with PD-1/PD-L1 inhibitor antibodies (ICIs), the risk of irAE is usually dose-dependent, with an incidence of 27% for all grades and 6% for grades ≥3 [72]. If, on the other hand, we consider the anti-CTLA-4 antibodies, the overall incidence of dose-dependent irAEs is considerably higher, reaching 72% for all grades and 24% for grade ≥3 [73,74]. These irAEs are usually easily managed by postponing the subsequent scheduled dose and using corticosteroids in the most severe cases and non-responders. A recent meta-analysis reports 42 (0.6%) cases of fatal irAEs amongst the 6528 patients treated with ICIs, and ipilimumab-induced colitis was the main cause of death [75]. Immune-related pneumonia [76] and myocarditis [77] may also have a fatal outcome. Despite this acceptable toxicity profile of ICIs in general, special attention must be exercised when they are administered to cirrhotic patients with HCC. This is, first and foremost, because the onset of immune-related hepatitis can cause an acute exacerbation of chronic liver failure with a high risk of severe hepatic decompensation and death due to the complications. Secondly, the use of corticosteroids as an antidote for the treatment of severe irAEs is particularly problematic in cirrhotic patients, especially in terms of an increase in the risk of infections and ascitic decompensation. Thirdly, cirrhosis is known to inhibit the homeostatic immune function of the liver, in itself resulting in a state of both systemic inflammation and immune deficiency [78]. Indeed, a study that enrolled patients treated with ICIs for different types of cancer suggests that hepatic AEs were associated with a worse prognosis [79]. Sangro et al. reported an aminotrasferase elevation rate of almost 50% in their pivotal study with tremelimumab. However, these changes were transient, never associated with hepatic impairment and resolved without the need for corticosteroids [80]. Fortunately, the subsequent clinical studies investigating the safety of ICIs in patients with HCC provided reassuring information [6,81,82]. Furthermore, the large-scale CheckMate 459 and KEYNOTE-240 studies confirmed that the safety profile of ICIs was consistent with that reported in previous studies for melanoma and lung cancer [13,83], suggesting that cirrhotic patients do not have an increased risk of hepatic irAEs. The percentage of cases requiring corticosteroid treatment was 6% in the study with durvalumab [82] and 20% for the nivolumab-ipilimumab combination [16]. It should be pointed out that the risk of serious irAEs in the studies on HCC increased when the ICIs were administered in combination with other cancer drugs. Overall, the data support the use of nivolumab even in Child-Pugh class B patients. In these particularly frail patients, treatment-related irAEs were reported in just 4 out of 49 patients and resulted in treatment discontinuation in 2 patients [7]. However, both HCC and liver cirrhosis can make recognising other irAEs more difficult. Skin toxicities, for example, are the most common irAEs reported in clinical studies with ICIs [16]. The interpretation of skin toxicities can be even more challenging when ICIs are administered in combination with TKIs, as this class of drugs is also potentially able to cause skin toxicity [84] such as HFSR, which is typical of TKIs. TKI-induced rash usually appears during the first week of treatment and tends to regress rapidly after treatment discontinuation, given the short half-life of most TKIs [9]. Skin toxicities associated with ICIs, on the other hand, tend to appear later and, in the absence of steroid therapy, their resolution can require prolonged treatment discontinuation [75]. Diarrhoea is another common irAE that it can be difficult to attribute to a precise cause. Indeed, cirrhotic patients are often treated with osmotic laxatives (the dose of which must be carefully adjusted) in order to prevent portosystemic encephalopathy. This symptom can regard patients treated with TKIs, ICIs or both. When diarrhoea is associated with abdominal pain and signs of colonic inflammation, immune-related colitis should be suspected and managed immediately, as it still represents the most common cause of death in patients treated with ICIs [75]. Although the diagnosis of immune-related colitis is often based on the presence of clinical signs and symptoms, colonoscopy remains the diagnostic gold standard for assessing severity and, therefore, also prognosis [85]. Immune-related endocrine disorders must also be thoroughly investigated. Thyroid function must always be monitored in patients with advanced HCC treated with ICIs and/or TKIs [3,9]. Consequently, immune-related hypo- and hyperthyroidism are usually diagnosed in the preclinical phase, whereas the identification of adrenal insufficiency can be more problematic, as cirrhotic patients have an intrinsic tendency towards hypotension due to the haemodynamic peculiarities of advanced liver disease, the mild hyponatraemia caused by haemodilution and usual use of potassium-sparing diuretics. Other irAEs, such as the onset of cough, fever and dyspnoea should be subject to immediate clinical investigations to exclude and/or confirm the suspicion of immune-related pneumonia that would require early treatment before it is able to cause acute respiratory insufficiency and, in some cases, also a negative prognosis [76].

## 9. Conclusions

In almost all cases, HCC develops in subjects with hepatic cirrhosis, often as the result of hepatitis B or C virus infection, alcohol abuse or metabolic forms secondary to non-alcoholic steatohepatitis. Patients with HCC and hepatic symptoms can therefore present symptoms that are attributable to both conditions. This creates a diverse stratification not only of the aetiological factors underlying the transformation process and their consequent management, but also of the treatment of the tumour and the factors influencing prognosis. The prognosis for HCC has changed greatly over the last two decades with the advent of novel treatment options associated with the screening programmes provided to patients with cirrhosis that make it possible to identify HCC at an early stage, and therefore to implement potentially increasingly curative treatments. The treatment options for HCC can be broken down into surgical therapies (i.e., resection, cryoablation and liver transplantation) and nonsurgical therapies that can target the liver and are therefore termed “locoregional”. After the failure of locoregional therapies, systemic treatment with Sorafenib has been the standard of care for advanced HCC for a long time. AEs commonly reported with TKIs include hypertension, diarrhoea, and HFSR and most AEs occur within the first month of treatment and resolve when treatment is put on hold. Patient education and frequent monitoring for symptoms are the key to appropriately managing TKI toxicities. The development of IO therapies for patients with HCC has advanced rapidly, and clinicians should be aware of the potential toxicities. The most common irAEs are similar to those with other tumour types, but the rate of hepatitis may be slightly higher. Most relevant, toxicities should be recognized early on and addressed appropriately. Recently, ICIs have shown potential in combination treatment for advanced HCC, although they have been quite unsuccessful as single agents. Some approaches attempted for use of ICIs in combination are anti-PD-L1 plus anti-VEGF (e.g., atezolizumab plus bevacizumab), anti-PD-1 plus TKI (e.g., pembrolizumab plus lenvatinib), anti-PD-L1 plus anti-CTLA-4 (e.g., nivolumab plus ipilimumab). In IMbrave 150 trial, the combination of atezolizumab plus bevacizumab has shown an OS advantage over TKI sorafenib, joining today this therapy as a frontline option for advanced HCC. For those who are not candidates for the atezolizumab—bevacizumab combination, either sorafenib or lenvatinib are appropriate alternative. Multiple ongoing trials with ICIs, VEGF inhibitors, and TKIs in the systemic treatment of advanced HCC promise expanding options for frontline and second-line therapies in HCC. These patients require a multidisciplinary management, especially calling for close interaction between the hepatologist and the oncologist. However, there are currently no biomarkers able to predict the toxicity of a given systemic treatment. A careful selection of patients based on their comorbidities and the functional status of their chronic liver disease therefore remains crucial. In order not to deprive patients of a systemic therapeutic option a priori, it is common clinical practice to adopt the empirical convention of initiating treatment with prudential doses of TKI before gradually increasing them in accordance with their tolerance.

## Figures and Tables

**Table 1:** Data from Child CG, Turcotte JG. Surgery and portal hypertension. In: Child CG. The liver and portal hypertension. Philadelphia: Saunders; **1964**.p.50–64.

**PARAMETER**	**1**	**2**	**3**
**Serum Bilirubin (mg/dL)**	**2.0**	2–3	>3.0
Serum Albumin (g/dL)	>3.515	2.8–3.5	<2.8
Prothrombin Time (Prolongation(s))	1–4	5–6	>6
Hepatic Encephalopathy	None	Minimal	Moderate
Ascites	None	Slight	Moderate
**1 and 2-years survival based on CTP Score Class**	**1 yr**	**2 yr**	
A (5–6 points)	100%	85%	
B (7–9 points)	80%	60%	
C (10–15 points)	45%	35%	

**Table 2:** Differences among locoregional therapies [38,39].

Treatment	Mortality	Major Complications	mOS	5 Years Survival
**RFA**	0.1–05%	2.2–4.1%	21 months	59.8%
**MWA**	0–0.36%	2.6–4.6%	31 months	67.9%
**PEI**	0.6	0–3.2%	NA	47–53%
**CA**	0%	2.8–3.9%	NA	59.5%
**IRE**	0%	3.3–4.7%	26.3	NA
**TACE**	1%	2.1–5%	38.3	27.6%
**DEB-TACE**	1.2%	3.2%	37	22.5%
**SIRT**	1.5%	4.9%	10–29	NA

mOS: median overall survival; RFA: radiofrequency thermal-ablation; MWA: microwave ablation; PEI: percutaneous ethanol injection; CA: cryoablation; IRE: irreversible electroporation; TAE: transarterial embolization; TACE: transarterial chemoembolization; DEB-TACE: transarterial chemoembolization with drug-eluting beads; SIRT: selective internal radiation therapy.

**Table 3:** Incidence of grade ≥3 AEs (%) occurring in ≥5% of HCC patients treated with TKIs [86].

Drug (Reference).	AE Grade ≥3.	Incidence ≥5 (%).
**Sorafenib** (3).	Diarrhoea.	11.
	Fatigue.	10.
	Abdominal pain.	9.
	HFSR.	8.
	Ascites.	7.
**Regorafenib** (5).	Hypertension.	15.
	HFSR.	13.
	Increased blood bilirubin.	11.
	Increased AST.	11.
	Fatigue.	9.
	Anaemia.	5..
**Lenvatinib** (9).	Hypertension.	23.
	Weight loss.	8.
	Increased blood bilirubin.	7.
	Proteinuria.	6.
	Decreased appetite.	5.
	Decreased platelet count.	5.
**Cabozantinib** (14).	HFSR.	17.
	Hypertension.	16.
	Increased AST.	12.
	Diarrhoea.	10.
	Fatigue.	10.
	Asthenia.	7.
	Decreased appetite.	6.
	Increased ALT.	5.

AE = adverse event; TKI = tyrosine kinase inhibitor; HFSR = hand-foot skin reaction; AST = aspartate transaminase; ALT = alanine aminotransferase.

**Table 4:** Essential guidance for patients initiating TKI therapy [86,87,88].

Provide the patient with adequate information on the potential adverse effects. Advise the patient to keep a diary in which to record his/her weight, blood pressure and bowel movements. Provide the patient with a list of products (creams containing urea, anti-diarrhoea products such as loperamide, anti-hypertensives: ACEi, ARB, beta blockers, non-dihydropyridine calcium-channel blockers) for the treatment and prevention of AEs. Instruct him/her on how to take the therapy. Provide information on any concomitant therapy considering potential drug-drug interactions (e.g., PPIs reduce the absorption of sorafenib by reducing gastric acidity). Provide practical information on the management of the most common adverse events (e.g., diarrhoea: avoid caffeine and spicy or fatty foods, dairy products, foods with a high fibre content and introduce potatoes, apple juice, probiotics, bananas and abundant oral hydration to prevent dehydration. For patients who frequently experienced diarrhoea, loperamide may also be taken pre-emptively. Concomitant lactulose dose reduction may be necessary. Loperamide-refractory diarrhoea may be treated with atropine-diphenoxylate, codeine or tincture of opium, if appropriate). Provide the patient with information on prophylactic management of HFSR (e.g., prophylactic use of emollients containing 10% urea and to remove existing areas of hyperkeratosis before TKI treatment initiation). Finally, in conventional clinical practice it is mandatory to establish early close follow-up after treatment initiation, and that patients should be given easy access to unscheduled visits and consultations to detect AEs, manage them promptly, and adjust dosage. This surely improves treatment compliance with optimal efficacy without unneeded treatment interruptions or cancellations.

TKIs = Tyrosine Kinase Inhibitors; ACEi = Angiotensin Converting Enzyme inhibitor; ARB = Angiotensin II Receptors Blocker; PPI = Proton Pump Inhibitor; HFSR = Hand-Foot Skin Reaction, AE = Adverse event.

**Table 5:** Safety data from clinical trials of immune checkpoint inhibitors in advanced HCC [89].

TOXICITIES.	Nivolumab.	Pembrolizumab.	Atezolizumab + Bevacizumab.	Pembrolizumab + Lenvatinib.	Nivolumab + Ipilimumab.
Grade ≥ 3 AEs.	22%.	46.3%.	56.5%.	67% (including three grade 5 events).	37%.
Discontinuation rate for AEs.	4%.	17.2%.	15.5%.	Not reported.	2–18%.

AE = adverse event; HCC = Hepatocellular carcinoma.

**Table 6:** Main AEs: Atezolizumab + Bevacizumab (AB) [12] vs. Pembrolizumab + Lenvatinib (PL) [69].

Adverse Event	AB (% Any Grade)	PL (% Any Grade)	≥Grade 3 (%) AB vs. PL
Hypertension	30	36	15 vs. 17
Fatigue	20	30	2 vs. 4
Proteinuria	20	20	3 vs. 4
AST increase	19.5	20	7 vs. 11
Pruritus	19.5	NR	0 vs. NR
Diarrhea	19	35	2 vs. 5
Decrease appetite	18	28	1 vs. 0
Pyrexia	18	NR	1 vs. NR
ALT increase	14	NR	1 vs. NR
Weight decrease	11	22	0 vs. 3
Constipation	13	NR	0 vs. NR
Nausea	12	17	0 vs. 1
Asthenia	7	19	0 vs. 5
Rash	12.5	NR	0 vs. NR
Hypothyroidism	NR	25	NR vs. 0
HFRS	1	23	0 vs. 1
Dysphonia	NR	21	NR vs. 1
Grade 5	4.6	13 *	4.6 vs. 13 *
**Total**	**98**	**99**	**56.5 vs. 67**

AE = adverse event; AST = aspartate aminotransferase; ALT = alanine aminotransferase; HFSR = hand-foot skin reaction; NR = not reported; * = n.3 deaths treatment-related.

## References

[1] The Cancer of the liver Italian Program (CLIP) investigators (1998). A new prognostic system for hepatocellular carcinoma: A retrospective study of 435 patients: The Cancer of the Liver Italian Program (CLIP) investigators. Hepatology.

[2] Younossi Z., Stepanova M., Ong J.P., Jacobson I.M., Bugianes E., Guseja A., Eguchi Y., Wong V.W., Negro F., Yilmaz Y. (2019). Non-alcoholic steato-hepatitis is the fastest growing cause of hepatocellular carcinoma in liver transplant candidates. Clin. Gastroenterol. Hepatol..

[3] Llovet J.M., Ricci S., Mazzaferro V., Hilgard P., Gane E., Blanc J.F., Cosme de Oliveira A., Santoro A., Raoul J.L., Forner A. (2008). Sorafenib in advanced hepatocellular carcinoma. N. Engl. J. Med..

[4] Cheng A.L., Kang Y.K., Chen Z., Tsao C.J., Qin S., Kim J.S., Luo R., Feng J., Shenglong Ye M.D., Yang T.S. (2009). Efficacy and safety of sorafenib in patients in the Asia-Pacific region with advanced hepatocellular carcinoma: A phase III randomised, double-blind, placebo-controlled trial. Lancet Oncol..

[5] Bruix J., Qin S., Merle P., Granito A., Huang Y.H., Bodoky G., Pracht M., Yokosuka O., Rosmorduc O., Breder M.D. (2017). RESORCE Investigators (2017). Regorafenib for patients with hepatocellular carcinoma who progressed on sorafenib treatment (RESORCE): A randomised, double-blind, placebo-controlled, phase 3 trial. Lancet.

[6] El-Khoueiry A.B., Sangro B., Yau T., Crocenzi T.S., Kudo M., Hsu C., Kim T.Y., Choo S.P., Trojan J., Welling T.H. (2017). Nivolumab in patients with advanced hepatocellular carcinoma (CheckMate 040): An open-label, non-comparative, phase 1/2 dose escalation and expansion trial. Lancet.

[7] Kudo M., Matilla A., Santoro A., Melero I., Gracian A.C., Rivera M.A., Choo S.P., El-Khoueiry A.B., Kuromatsu R., El-Rayes B.F. (2019). Checkmate-040: Nivolumab (NIVO) in patients (pts) with advanced hepatocellular carcinoma (aHCC) and Child-Pugh B (CPB) status. J. Clin. Oncol..

[8] Kambhampati S., Bauer K.E., Bracci P.M., Keenan B.P., Behr S.C., Gordan J.D., Kelley R.K. (2019). Nivolumab in patients with advanced hepatocellular carcinoma and Child-Pugh class B cirrhosis: Safety and clinical outcomes in a retrospective case series. Cancer.

[9] Kudo M., Finn R.S., Qin S., Han K.H., Ikeda K., Piscaglia F., Baron A., Park J.W., Han G., Jassem J. (2018). Lenvatinib versus sorafenib in first-line treatment of patients with unresectable hepatocellular carcinoma: A randomised phase 3 non-inferiority trial. Lancet.

[10] (2020). NCCN Clinical Practice Guidelines in Oncology: Hepatobiliary Cancers v5.

[11] Alsina A., Kudo M., Vogel A., Cheng A.L., Tak W.Y., Ryoo B.Y., Evans T., López López C., Daniele B., Misir S. (2019). Effects of Subsequent Systemic Anticancer Medication Following First-Line Lenvatinib: A Post Hoc Responder Analysis from the Phase 3 REFLECT Study in Unresectable Hepatocellular Carcinoma. Liver Cancer.

[12] Finn R.S., Qin S., Ikeda M., Galle P.R., Ducreux M., Kim T.Y., Kudo M., Breder V., Merle P., Kaseb A.O. (2020). Atezolizumab plus Bevacizumab in Unresectable Hepatocellular Carcinoma. N. Engl. J. Med..

[13] Yau T., Park J.W., Finn R.S., Cheng A.L., Mathurin P., Edeline J., Kudo M., Han K.H., Harding J.J., Merle P. (2019). CheckMate 459: A randomized, multi-center phase III study of nivolumab (NIVO) vs. sorafenib (SOR) as first-line (1L) treatment in patients (pts) with advanced hepatocellular carcinoma. Ann. Oncol..

[14] Abou-Alfa G.K., Meyer T., Cheng A.L., El-Khoueiry A.B., Rimassa L., Ryoo B.Y., Cicin I., Merle P., Chen Y., Park J. (2018). Cabozantinib in Patients with Advanced and Progressing Hepatocellular Carcinoma. N. Engl. J. Med..

[15] Zhu A.X., Kang Y.K., Yen C.J., Finn R.S., Galle P.R., Llovet J.M., Assenat E., Brandi G., Pracht M., Lim H.Y. (2018). REACH-2: A randomized, double-blind, placebo-controlled phase 3 study of ramucirumab versus placebo as second-line treatment in patients with advanced hepatocellular carcinoma (HCC) and elevated baseline alpha-fetoprotein (AFP) following first-line sorafenib. J. Clin. Oncol..

[16] Yau T., Kang Y., Kim T., El-Khoueiry A.B., Santoro A., Sangro B., Melero I., Kudo M., Hou M., Matilla A. (2019). Nivolumab + ipilimumab combination therapy in patients with advanced hepatocellular carcinoma: Results from CheckMate 040. J. Clin. Oncol..

[17] Zhu A.X., Finn R.S., Edeline J., Cattan S., Ogasawara S., Palmer D., Verslype C., Zagonel V., Fartoux L., Vogel A. (2018). Pembrolizumab in patients with advanced hepatocellular carcinoma previously treated with sorafenib (KEYNOTE-224): A non- randomised, open-label phase 2 trial. Lancet Oncol..

[18] Llovet J.M., Bru C., Bruix J. (1999). Prognosis of hepatocellular carcinoma: The BCLC staging classification. Semin. Liver Dis..

[19] Kamath P.S., Wiesner R.H., Malinchoc M., Kremers W., Therneau T.M., Kosberg C.L., D’Amico G., Dickson E.R., Kim W.R. (2001). A model to predict survival in patients with end-stage liver disease. Hepatology.

[20] Johnson P.J., Berhane S., Kagebayashi C., Satomura S., Teng M., Reeves H.L., O’Beirne J., Fox R., Skowronska A., Palmer D. (2015). Assessment of liver function in patients with hepatocellular carcinoma: A new evidence-based approach-the ALBI grade. J. Clin. Oncol..

[21] European Association for the Study of the Liver (2018). Electronic address: easloffice@easloffice.eu, & European Association for the Study of the Liver. J. Hepatol..

[22] Mazzaferro V., Regalia E., Doci R., Andreola S., Pulvirenti A., Bozzetti F., Montalto F., Ammatuna M., Morabito A., Gennari L. (1996). Liver transplantation for the treatment of small hepatocellularcarcinomas in patients with cirrhosis. N. Engl. J. Med..

[23] Marrero J.A., Kulik L.M., Sirlin C.B., Zhu A.X., Finn R.S., Abecassis M.M., Roberts L.R., Heimbac J.K. (2018). Diagnosis, staging, and management of Hepatocellular Carcinoma: 2018 practice guidance by the American Association for the Study of Liver Diseases. Hepatology.

[24] Miguet M., Adam J.P., Blanc J.F., Lapuyade B., Bernard P., Buscail E., Neau-Cransac M., Vendrely V., Laurent C., Chiche L. (2019). Multidisciplinary meetings specific to hepatocellular carcinoma: How to proceed?. J. Visc. Surg..

[25] Barone C., Koeberle D., Metselaar H., Parisi G., Sansonno D., Spinzi G. (2013). Multidisciplinary approach for HCC patients: Hepatology for the oncologists. Ann. Oncol..

[26] Karnofsky D., Burchenal J., MacLeod C. (1949). The clinical evaluation of chemotherapeutic agents in cancer. Evaluation of Chemotherapeutic Agents.

[27] Oken M.M., Creech R.H., Tormey D.C., Horton J., Davis T.E., McFadden E.T., Carbone P.P. (1982). Toxicity And Response Criteria Of The Eastern Cooperative Oncology Group. Am. J. Clin. Oncol..

[28] Child C.G., Turcotte J.G. (1964). Surgery and portal hypertension. Major Probl. Clin. Surg..

[29] Farinati F., Vitale A., Spolverato G., Pawlik T.M., Huo T.L., Lee Y.H., Frigo A.C., Giacomin A., Giannini E.G., Ciccarese F. (2016). Development and Validation of a New Prognostic System for Patients with Hepatocellular Carcinoma. PLoS Med..

[30] Borzio M., Dionigi E., Rossini A., Marignani M., Sacco R., De Sio I., Bertolini E., Francica G., Giacomin A., Parisi G. (2018). External validation of the ITA.LI.CA prognostic system for patients with hepatocellular carcinoma: A multicenter cohort study. Hepatology.

[31] Durand F., Valla D. (2005). Assessment of the prognosis of cirrhosis: Child-Pugh versus MELD. J. Hepatol..

[32] Ripoll C., Genescà J., Araujo I.K., Graupera I., Augustin S., Tejedor M., Cirera I., Aracil C., Sala M., Hernandez-Guerra M. (2013). Rebleeding prophylaxis improves outcomes in patients with hepatocellular carcinoma. A multicenter case-control study. Hepatology.

[33] Tamaoki M., Toshikuni N., Matsueda K., Yamamoto H. (2012). Influence of high-risk esophageal varices on outcomes in hepatocellular carcinoma patients: Benefits of prophylactic endoscopic therapies. Hepatogastroenterology.

[34] Giovanardi F., Lai Q., Bertacco A., Vitale A. (2018). Resection for hepatocellular cancer: Overpassing old barriers. Transl. Gastroenterol. Hepatol..

[35] Majno P.E., Mentha G., Mazzaferro V. (2010). Partial hepatectomy versus radiofrequency ablation for hepatocellular carcinoma: Confirming the trial that will never be, and some comments on the indications for liver resection. Hepatology.

[36] Vitale A., Peck-Radosavljevic M., Giannini E.G., Vibert E., Sieghart W., Van Poucke S., Pawlik T.M. (2017). Personalized treatment of patients with very early hepatocellular carcinoma. J. Hepatol..

[37] Cucchetti A., Sposito C., Pinna A.D., Citterio D., Ercolani G., Flores M., Cescon M., Mazzaferro V. (2016). Effect of age on survival in patients undergoing resection of hepatocellular carcinoma. Br. J. Surg..

[38] Chen M.S., Li J.Q., Zheng Y., Guo R.P., Liang H.H., Zhang Y.Q., Lin X.J., Lau W.Y. (2006). A prospective randomized trial comparing percutaneous local ablative therapy and partial hepatectomy for small hepatocellular carcinoma. Ann. Surg..

[39] Marelli L., Stigliano R., Triantos C., Senzolo M., Cholongitas E., Davies N., Yu D., Meyer T., Patch D.W., Burroughs A.K. (2006). Treatment outcomes for hepatocellular carcinoma using chemoembolization in combination with other therapies. Cancer Treat. Rev..

[40] Cho Y.K., Kim J.K., Kim W.T., Chung J.W. (2010). Hepatic resection versus radiofrequency ablation for very early stage hepatocellular carcinoma: A Markov model analysis. Hepatology.

[41] Golfieri R., Bilbao J.I., Carpanese L., Cianni R., Gasparini R., Ezziddin S., Paprottka P.M., Fiore F., Cappelli A., Rodriguez M. (2013). Comparison of the survival and tolerability of radioembolization in elderly vs. younger patients with unresectable hepatocellular carcinoma. J. Hepatol..

[42] Chinnaratha M.A., Chuang M.Y., Fraser R.J., Woodman R.J., Wigg A.J. (2016). Percutaneous thermal ablation for primary hepatocellular carcinoma: A systematic review and meta-analysis. J. Gastroenterol. Hepatol..

[43] Tan W., Deng Q., Lin S., Wang Y., Xu G. (2019). Comparison of microwave ablation and radiofrequency ablation for hepatocellular carcinoma: A systematic review and meta-analysis. Int. J. Hyperthermia.

[44] Vietti Violi N., Duran F., Guiu B., Cercueil J.P., Aubé C., Digklia A., Pache I., Deltenre P., Knebel J.F., Denys A. (2018). Efficacy of microwave ablation versus radiofrequency ablation for the treatment of hepatocellular carcinoma in patients with chronic liver disease: A randomised controlled phase 2 trial. Lancet Gastroenterol. Hepatol..

[45] Germani G., Pleguezuelo M., Gurusamy K., Meyer T., Isgrò G., Burroughs A.K. (2010). Clinical outcomes of radiofrequency ablation, percutaneous alcohol and acetic acid injection for hepatocellular carcinoma: A meta-analysis. J. Hepatol..

[46] Chen Q.W., Ying H.F., Gao S., Shen Y.-H., Meng Z.-Q., Chen H., Chen Z., Tenget W.-J. (2016). Radiofrequency ablation plus chemoembolization versus radiofrequency ablation alone for hepatocellular carcinoma: A systematic review and meta-analysis. Clin. Res. Hepatol. Gastroenterol..

[47] Rajyaguru D.J., Borgert A.J., Smith A.L., Thomes R.M., Conway P.D., Halfdanarson T.R., Truty M.J., Kurup N., Go R.S. (2018). Radiofrequency ablation versus stereotactic body radiotherapy for localized hepatocellular carcinoma in nonsurgically managed patients: Analysis of the national cancer database. J. Clin. Oncol..

[48] Xie H., Yu H., Tian S., Yang X., Wang X., Yang Z., Wang H., Guo Z. (2018). What is the best combination treatment with transarterial chemoembolization of unresectable hepatocellular carcinoma? A systematic review and network meta-analysis. Oncotarget.

[49] Sacco R., Bargellini I., Bertini M., Bozzi E., Romano A., Petruzzi P., Tumino E., Ginanni B., Federici G., Cioni R. (2011). Conventional versus doxorubicin-eluting bead transarterial chemoembolization for hepatocellular carcinoma. J. Vasc. Interv. Radiol..

[50] Golfieri R., Giampalma E., Renzulli M., Cioni R., Bargellini I., Bartolozzi C., Breatta A.D., Gandini G., Nani R., Gasparini D. (2014). Randomised controlled trial of doxorubicin-eluting beads vs. conventional chemoembolisation for hepatocellular carcinoma. Br. J. Cancer.

[51] Lencioni R., Llovet J.M. (2010). Modified RECIST (mRECIST) assessment for hepatocellular carcinoma. Semin. Liver Dis..

[52] Piscaglia F., Ogasawara S. (2018). Patient Selection for Transarterial Chemoembolization in Hepatocellular Carcinoma: Importance of Benefit/Risk Assessment. Liver Cancer.

[53] Ishikawa T. (2018). Prevention of post-embolization syndrome after transarterial chemoembolization for hepatocellular carcinoma—Is prophylactic dexamethasone useful, or not?. Hepatobiliary Surg. Nutr..

[54] Miksad R.A., Ogasawara S., Xia F., Fellous M., Piscaglia F. (2019). Liver function changes after transarterial chemoembolization in US hepatocellular carcinoma patients: The LiverT study. BMC Cancer.

[55] Edeline J., Crouzet L., Campillo-Gimenez B., Rolland Y., Pracht M., Guillygomarc’h A., Boudjema K., Lenoir L., Adhoute X., Rohou T. (2016). Selective internal radiation therapy compared with sorafenib for hepatocellular carcinoma with portal vein thrombosis. Eur. J. Nucl. Med. Mol. Imaging.

[56] Sangro B., Maini C.L., Ettorre G.M., Cianni R., Golfieri R., Gasparini D., Ezziddin S., Paprottka P.M., Fiore F., Van Buskirk M. (2018). Radioembolisation in patients with hepatocellular carcinoma that have previously received liver-directed therapies. European Network on Radioembolization with Yttrium-90 resin microspheres (ENRY). Eur. J. Nucl. Med. Mol. Imaging.

[57] Vilgrain V., Pereira H., Assenat E., Guiu B., Ilonca A.D., Pageaux G.-P., Sibert A., Bouattour M., Lebtahi R., Allaham W. (2017). SARAH Trial Group. Efficacy and safety of selective internal radiotherapy with yttrium-90 resin microspheres compared with sorafenib in locally advanced and inoperable hepatocellular carcinoma (SARAH): An open-label randomised controlled phase 3 trial. Lancet Oncol..

[58] Chow P.K.H., Gandhi M., Choo S.P., Thng C.H., Tan S.B., Low A.S.C., Cheow P.C., Goh A.S.W., Tay K.H., Lo R.H.G. (2017). Phase III multi-centre open-label randomized controlled trial of selective internal radiation therapy (SIRT) versus sorafenib in locally advanced hepatocellular carcinoma: The SIRveNIB study. 2017 ASCO Annual Meeting. J. Clin. Oncol..

[59] Hilgard P., Hamami M., Fouly A.E., El Fouly A., Scherag A., Müller S., Ertle J., Heusner T., Cicinnati V.R., Paul A. (2010). Radioembolization with yttrium-90 glass microspheres in hepatocellular carcinoma: European experience on safety and long-term survival. Hepatology.

[60] Riaz A., Lewandowski R.J., Kulik L.M., Mulcahy M.F., Sato K.T., Ryu R.K., Omary R.A., Salem R. (2009). Complications following radioembolization with yttrium-90 microspheres: A comprehensive literature review. J. Vasc. Interv. Radiol..

[61] Gil-Alzugaray B., Chopitea A., Iñarrairaegui M., Bilbao J.I., Rodriguez-Fraile M., Rodriguez J., Benito A., Dominguez I., D’Avola D., Herrero J.I. (2013). Prognostic factors and prevention of radioembolization-induced liver disease. Hepatology.

[62] Papatheodoridis G., Dalekos G., Sypsa V., Yurdaydin C., Buti M., Goulis J., Calleja J.L., Chi H., Manolakopoulos S., Mangia G. (2016). PAGE-B predicts the risk of developing hepatocellular carcinoma in Caucasians with chronic hepatitis B on 5-year antiviral therapy. J. Hepatol..

[63] Cabibbo G., Petta S., Barbara M., Attardo S., Bucci L., Farinati F., Giannini E.G., Negrini G., Ciccarese F., Rapaccini G.L. (2017). Hepatic decompensation is the major driver of death in HCV-infected cirrhotic patients with successfully treated early hepatocellular carcinoma. J. Hepatol..

[64] Cabibbo G., Celsa C., Calvaruso V., Petta S., Cacciola I., Cannavò M.R., Madonia S., Rossi M., Magro B., Rini F. (2019). Direct-acting antivirals after successful treatment of early hepatocellular carcinoma improve survival in HCV-cirrhotic patients. J. Hepatol..

[65] Arora S.P., Liposits G., Caird S., Dunne R.F., Moffat G.T., Okonji D., Rodriquenz M.G., Dua D., Dotan E. (2020). Hepatocellular carcinoma in older adults: A comprehensive review by Young International Society of Geriatric Oncology. J. Geritar. Oncol..

[66] Arora S.P., Ketchum N.S., Gelfond J. (2018). Comparative efficacy and safety of sorafenib in elderly versus non-elderly patients with advanced hepatocellular carcinoma (HCC) with varying liver dysfunction. J. Clin. Oncol..

[67] Di Costanzo G.G., Tortora R., De Luca M., Galeota Lanza A., Lampasi F., Tartaglione M.T., Picciotto F.P., Imparato M., Mattera S., Cordone G. (2013). Impact of age on toxicity and efficacy of sorafenib-targeted therapy in cirrhotic patients with hepatocellular carcinoma. Med. Oncol..

[68] Williet N., Clavel L., Bourmaud A., Verot C., Bouarioua N., Roblin X., Merle P., Jean-Marc Phelip J.-M. (2017). Tolerance and outcomes of sorafenib in elderly patients treated for advanced hepatocellular carcinoma. Dig. Liver Dis..

[69] Finn R.S., Ikeda M., Zhu A.X., Sung M.W., Baron A.D., Kudo M., Okusaka T., Kobayashi M., Kumada H., Kaneko S. (2020). Phase Ib study of Lenvatinib plus pembrolizumab in patients with unresectable hepatocellular carcinoma. J. Clin. Oncol..

[70] Wong H., Tang Y.F., Yao T.J., Chiu J., Leung R., Chan P., Cheung T.T., Chan A.C., Pang R.W., Poon R. (2011). The outcomes and safety of single-agent sorafenib in the treatment of elderly patients with advanced hepatocellular carcinoma (HCC). Oncologist.

[71] Collins B.H., Pirsch J.D., Becker Y.T., Hanaway M.J., Van der Werf W.J., D’Alessandro A.M., Knechtle S.J., Odorico J.S., Leverson G., Musat A. (2000). Long-term results of liver transplantation in older patients 60 years of age and older. Transplantation.

[72] Majumdar A., Roccarina D., Thorburn D., Davidson B.R., Tsochatzis E., Gurusamy S.G. (2017). Management of people with early- or very early-stage hepatocellular carcinoma: An attempted network meta-analysis. Cochrane Database Syst. Rev..

[73] Wang P.F., Chen Y., Song S.Y., Wang T.J., Ji W.-J., Li S.-W., Liu N., Yan C.-X. (2017). Immune-Related Adverse Events Associated with Anti-PD-1/PD-L1 Treatment for Malignancies: A Meta-Analysis. Front. Pharm..

[74] Bertrand A., Kostine M., Barnetche T., Truchetet M.-E., Schaeverbeke T. (2015). Immune related adverse events associated with anti-CTLA-4 antibodies: Systematic review and meta-analysis. BMC Med..

[75] De Velasco G., Je Y., Bossé D., Awad M.M., Ott P.A., Moreira R.B., Schutz F., Bellmunt J., Sonpavde G.P., Hodi F.S. (2017). Comprehensive Meta-analysis of Key Immune-Related Adverse Events from CTLA-4 and PD-1/PD-L1 Inhibitors in Cancer Patients. Cancer Immunol. Res..

[76] Abdel-Rahman O., Fouad M. (2016). Risk of pneumonitis in cancer patients treated with immune checkpoint inhibitors: A meta-analysis. Adv. Respir. Dis..

[77] Johnson D.B., Balko J.M., Compton M.L., Chalkias S., Gorham J., Xu Y., Hicks M., Puzanov I., Alexander M.R., Bloomer T.L. (2016). Fulminant Myocarditis with Combination Immune Checkpoint Blockade. N. Engl. J. Med..

[78] Albillos A., Lario M., Álvarez-Mon M. (2014). Cirrhosis-associated immune dysfunction: Distinctive features and clinical relevance. J. Hepatol..

[79] Chan S.L., Yip T.C.-F., Wong V.W.-S., Tse Y.-K., Yuen B.W.-Y., Luk H.W.-S., Lui R.N.-S., Chan H.L.-Y., Mok T.S.-K., Wong G.L.-H. (2020). Pattern and impact of hepatic adverse events encountered during immune checkpoint inhibitors—A territory-wide cohort study. Cancer Med..

[80] Sangro B., Gomez-Martin C., de la Mata M., Iñarrairaegui M., Garralda E., Barrera P., Riezu-Boj J.I., Larrea E., Alfaro C., Sarobe P. (2013). A clinical trial of CTLA-4 blockade with tremelimumab in patients with hepatocellular carcinoma and chronic hepatitis C. J. Hepatol..

[81] Qin S., Ren Z., Meng Z., Chen Z., Chai X., Xiong J., Bai Y., Yang L., Zhu H., Fang W. (2020). Camrelizumab in patients with previously treated advanced hepatocellular carcinoma: A multicentre, open-label, parallel-group, randomised, phase 2 trial. Lancet Oncol..

[82] Wainberg Z.A. (2017). Safety and Clinical Activity of Durvalumab Monotherapy in Patients with Hepatocellular Carcinoma (HCC). J. Clin. Oncol..

[83] Finn R.S., Ryoo B.-Y., Merle P., Kudo M., Bouattour M., Lim H.Y., Breder V., Edeline J., Chao Y., Ogasawara S. (2020). Pembrolizumab As Second-Line Therapy in Patients With Advanced Hepatocellular Carcinoma in KEYNOTE-240: A Randomized, Double-Blind, Phase III Trial. J. Clin. Oncol..

[84] Tovoli F., Ielasi L., Casadei-Gardini A., Granito A., Foschi F.G., Rovesti G., Negrini G., Orsi G., Renzulli M., Piscaglia F. (2019). Management of adverse events with tailored sorafenib dosing prolongs survival of hepatocellular carcinoma patients. J. Hepatol..

[85] Prieux-Klotz C., Dior M., Damotte D., Dreanic J., Brieau B., Brezault C., Abitbol V., Chaussade S., Coriat R. (2017). Immune Checkpoint Inhibitor-Induced Colitis: Diagnosis and Management. Target Oncol..

[86] Rimassa L., Danesi R., Pressiani T., Merle P. (2019). Management of adverse events associated with tyrosine kinase inhibitors: Improving outcomes for patients with hepatocellular carcinoma. Cancer Treat. Rev..

[87] Eso Y., Marusawa H. (2018). Novel approaches for molecular targeted therapy against hepatocellular carcinoma. Hepatol. Res..

[88] Reig M., Torres F., Rodriguez-Lope C., Forner A., Llarch N., Rimola J., Darnell A., Rios J., Ayuso C., Bruix J. (2014). Early dermatologic adverse events predict better outcome in HCC patients treated with sorafenib. J. Hepatol..

[89] Tovoli F., De Lorenzo S., Trevisani F. (2020). Immunotherapy with checkpoint inhibitors for hepatocellular carcinoma: Where are we now?. Vaccines.

